# Systematic Identification of Methyl Jasmonate-Responsive Long Noncoding RNAs and Their Nearby Coding Genes Unveils Their Potential Defence Roles in Tobacco BY-2 Cells

**DOI:** 10.3390/ijms232415568

**Published:** 2022-12-08

**Authors:** Kaifeng Zheng, Zitao Wang, Lu Pang, Zhongbang Song, Heping Zhao, Yingdian Wang, Bingwu Wang, Shengcheng Han

**Affiliations:** 1 Beijing Key Laboratory of Gene Resources and Molecular Development, College of Life Sciences, Beijing Normal University, Beijing 100875, China; 2 College of Life Sciences, Qinghai Normal University, Xining 810008, China; 3 Academy of Plateau Science and Sustainability of the People’s Government of Qinghai Province & Beijing Normal University, Qinghai Normal University, Xining 810008, China; 4 Yunnan Academy of Tobacco Agricultural Sciences, Kunming 650021, China

**Keywords:** long noncoding RNA, methyl jasmonate, tobacco, nicotine synthesis, plant defence

## Abstract

Long noncoding RNAs (lncRNAs) are distributed in various species and play critical roles in plant growth, development, and defence against stimuli. However, the lncRNA response to methyl jasmonate (MeJA) treatment has not been well characterized in *Nicotiana tabacum* Bright Yellow-2 (BY-2) cells, and their roles in plant defence remain elusive. Here, 7848 reliably expressed lncRNAs were identified in BY-2 cells, of which 629 differentially expressed (DE) lncRNAs were characterized as MeJA-responsive lncRNAs. The lncRNAs in BY-2 cells had a strong genus specificity in *Nicotiana*. The combined analysis of the *cis*-regulated lncRNAs and their target genes revealed the potential up- and downregulated target genes that are responsible for different biological functions and metabolic patterns. In addition, some lncRNAs for response-associated target genes might be involved in plant defence and stress resistance via their MeJA- and defence-related *cis*-regulatory elements. Moreover, some MeJA-responsive lncRNA target genes were related to quinolinate phosphoribosyltransferase, lipoxygenases, and endopeptidase inhibitors, which may contribute to nicotine synthesis and disease and insect resistance, indicating that MeJA-responsive lncRNAs regulate nicotine biosynthesis and disease resistance by regulating their potential target genes in BY-2 cells. Therefore, our results provide more targets for genetically engineering the nicotine content and plant defence in tobacco plants.

## 1. Introduction

With the development of tiling arrays and next-generation sequencing (NGS), noncoding RNAs (ncRNAs) are being recognized as an increasingly important part of the transcriptomes of eukaryotic organisms [1,2]. Small RNAs [18–30 nucleotides (nt)], medium-sized ncRNAs (31–200 nt), and long ncRNAs (>200 nt) are the three major types of ncRNAs [3]. Among them, long noncoding RNAs (lncRNAs) have recently emerged as powerful and essential function regulators, notably in mammals [4,5,6]. However, research on diverse and complex lncRNAs in plants is still in its early stages. Small interfering RNA (siRNA) precursors, scaffolds, and molecular sponges are several functions of plant lncRNAs induced by various environmental conditions [3,7]. Some plant lncRNAs, such as *COLDAIR* and *COOLAIR*, can modulate transcription via chromatin changes and become scaffolds of DNA or protein [8,9]. Several lncRNAs function as decoys that alter target protein behaviour. During developmental transitions, *Arabidopsis ASCO*-lncRNA competes with NUCLEAR SPECKLE RNA-BINDING PROTEINS (NSRs) to modulate alternative splicing and gene expression by binding to the NSRs, the splicing factors [10]. It was discovered, for the first time, that *ENOD40* encodes the functional peptides in *Medicago sativa* and soybean, and the peptides encoded by lncRNAs can sometimes be essential for lncRNA function [11,12]. In addition, lncRNAs can respond to biotic or abiotic stresses, such as pathogen infection and extreme temperatures, and can stratify their expression spatially and temporally to combat diverse stress conditions as response effectors [13].

Plants have evolved various defences to reduce the risk of harm and productivity loss in response to attacks [14]. Plant defence has been divided into tolerance and resistance strategies, which can include deterring pest-landing, preventing feeding, and reducing palatability [15,16,17]. Whether attacked by pathogens, insects, herbivores, or even other abiotic stresses, a wide range of sophisticated molecular mechanisms allow plants to respond and resist [14]. Plants can generate a wide range of secondary metabolites, including crucial chemicals, such as alkaloids, which are primarily used to attract pollinating insects and defend against diseases and herbivores [18,19]. Since the beginning of human civilization, alkaloid-containing plant extracts have served as poisons, drugs, and potential medicines [20]. Tobacco (*Nicotiana tabacum*) is a species of the Solanaceae (nightshade) family that has been discovered to produce alkaloids and has become a commercially crucial agricultural plant [20,21].

As leaves are attacked, alkaloid synthesis is mainly triggered by jasmonate (JA) signalling in the root, which is tightly regulated by transcription [22,23]. The jasmonates, including jasmonic acid and its derivatives, are phytohormones derived from oxylipins that regulate plant growth, development, reproduction, and defence [24]. Endogenous JA levels in plants have been demonstrated to rise rapidly in response to wounding and other stressors, and nicotine levels can be increased with exogenous methyl jasmonate (MeJA) [25,26,27]. MeJA is used in experiments to mimic herbivory and induce plant defences, and it provides us with an approach to studying the molecular mechanisms of plant defence [28]. As everyone knows, the coordinated action of v-myb avian myeloblastosis viral oncogene homolog (MYB)- and basic helix-loop-helix (bHLH)-type TFs modulates flavonoid biosynthesis [29,30]. In *Catharanthus roseus*, the APETALA 2/ethylene response factor (AP2/ERF)–domain transcription factors could regulate terpenoid indole alkaloid biosynthesis [31,32,33,34]. Of course, countless metabolic processes are involved in plant defence and stress, and above, we have described just one of the metabolites that have received the most attention [35].

However, the molecular identification of lncRNAs in plant stress, disease resistance, and defence remains a great mystery. The *N. tabacum* genome is complex and difficult to portray because it is an allotetraploid, most likely the result of hybridization between diploid *N. sylvestris* and *N. tomentosiformis* ancestors [36,37]. Not only do hybrid offspring outperform their parents in terms of phenotypic traits, but polyploidy plants contain more functional genes and complex gene regulatory networks [38,39,40]. In this study, we first systematically identified MeJA-responsive lncRNAs in tobacco BY-2 cells from previously performed RNA-seq, investigated their evolutionary relationship, predicted their potential target genes, and analysed their functions associated with plant defence. Overall, the results of our study increased insight into MeJA-responsive lncRNAs in tobacco BY-2 cells and provided an abundant resource for further research on these MeJA-responsive lncRNAs associated with plant defence.

## 2. Results

### 2.1. Reliable Mining for lncRNAs Indicates That Intergenic Transcripts Are the Most Prominent Group

**Figure 1:**
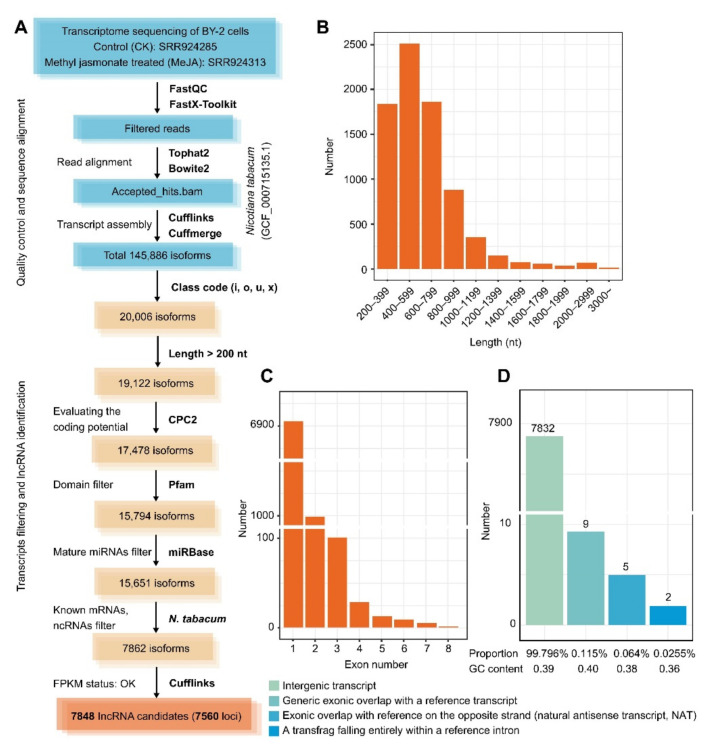
Identification and characteristics of tobacco long noncoding RNAs (lncRNAs) expressed in BY-2 cells. (**A**) General RNA-seq processing and lncRNA identification pipeline in tobacco BY-2 cells. (**B**) Length distribution of 7848 lncRNAs in tobacco BY-2 cells. (**C**) Distribution of exon numbers in tobacco BY-2 cells. (**D**) Classification of lncRNAs according to their location on the genome in relation to neighbouring genes. The columns indicate the number of lncRNAs for each class. The proportions and GC content of the four lncRNA kinds were calculated.

After the read alignment, transcript assembly, and systematic exploration of the lncRNAs, we identified 7848 lncRNAs derived from 7560 loci based on RNA-seq datasets from MeJA-untreated and MeJA-treated BY-2 cells (Figure 1A and Supplementary Table S1). It should be noted that each group of the reading segment data matched the tobacco genome at a rate of approximately 95%, and all of the lncRNAs that were eventually examined had realistic FPKM values. We know that the length of lncRNAs is generally greater than 200 nucleotides (nt), and therefore, we focused on the length distribution of the lncRNAs. In the BY-2 cells, the lncRNA transcript lengths varied from 201 to 5132 nt, with a mean of 631 nt. We found that lncRNAs of 400–599 nt were the most abundant (*n* = 2510), while the number of lncRNAs ranging from 200–399 nt and 600–799 nt was quite close (Figure 1B and Supplementary Table S2). Regarding the exon count, 6971 lncRNAs had one exon, and 975 lncRNAs had two exons. The presence of multiple exons (from three to eight) was not the primary feature of tobacco BY-2 cell lncRNAs (Figure 1C and Supplementary Table S3). Furthermore, just one lncRNA contained eight exons, the most exons of any lncRNA. Then, we divided the lncRNAs into four primary types based on their location in the genome with neighbouring genes (Figure 1D). In detail, there were 7832 intergenic transcripts, nine transcripts of generic exonic overlap with reference transcripts, five transcripts of exonic overlap with reference on the opposite strand, and two transcripts of transfrag falling entirely within the reference intron. Therefore, the intergenic lncRNAs appeared to make up the majority of the lncRNAs (99.796%), which had an average GC content of 0.39, close to the genomic sequence of *N. tabacum* (0.39) (Figure 1D).

### 2.2. The lncRNAs in BY-2 Cells Have Extreme Genus Specificity for Nicotiana

**Figure 2:**
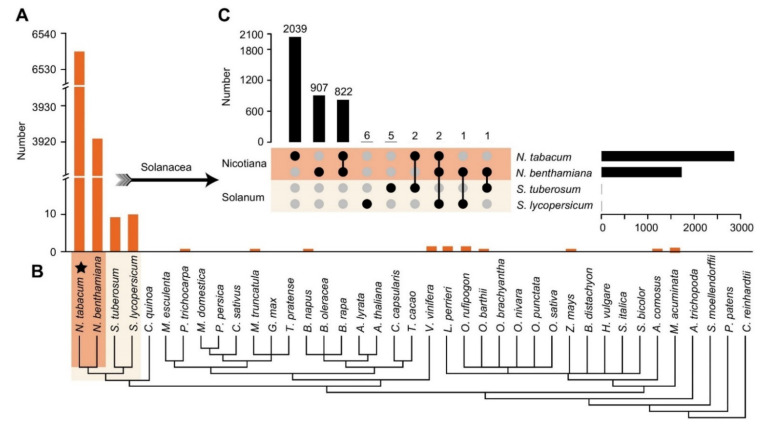
Comparison of conservation of lncRNAs in tobacco BY-2 cells with those in 39 other plants. (**A**) The bar chart in orange shows the number of lncRNAs in every examined plant homologous to those in tobacco BY-2 cells. (**B**) Phylogenetic relationships of 39 plants in which Solanaceae (containing *Nicotiana* and *Solanum*) are highlighted with color blocks. Asterisks indicate the paralogous or the same lncRNAs between *N. tabacum* and BY-2 cells. (**C**) The specific and common lncRNAs in four Solanaceae plants homologous to those in tobacco BY-2 cells.

To comprehensively investigate the evolutionary relationship of lncRNAs in tobacco BY-2 cells, we retrieved the lncRNAs and phylogenetic relationships of thirty-nine species, including eudicots (twenty), monocots (fourteen), basal angiosperms (one), ferns (one), mosses (one), and green algae (one) (Figure 2B). Using tobacco BY-2 cells as an example, only 4580 (58.359%) of the 7848 lncRNAs were homologous or paralogous with those of the above species (Figure 2A and Supplementary Tables S4 and S5). Within the four Solanaceae species, there was a relatively high sequence similarity, with 6535 (83.269%), 3921 (49.962%), 9 (0.115%), and 10 (0.127%) lncRNAs in *N. tabacum*, *N. benthamiana*, *S. tuberosum*, and *S. lycopersicum* having homology with tobacco BY-2 cells, respectively. It should be noted that the BY-2 cells were derived from *N. tabacum*, and therefore 6535 lncRNAs were paralogous or the same lncRNAs. Furthermore, we found that 822 lncRNAs of tobacco BY-2 cells were homologous between *N. tabacum* and *N. benthamiana*, indicating that they might be highly conserved in *Nicotiana* (Figure 2C). The lncRNAs in the BY-2 cells were approximately one-fourth of all the lncRNAs in *N. tabacum* (31,028). The maximum percentage of the homologous was only 0.025% in other non-Solanaceae, and no homologous lncRNAs were detected in most of the species. These findings suggested that lncRNAs in tobacco BY-2 cells were poorly conserved with most plants but had extreme genus specificity in *Nicotiana*.

### 2.3. The Upregulated lncRNAs Constitute a Large Majority of the MeJA-Responsive lncRNAs

**Figure 3:**
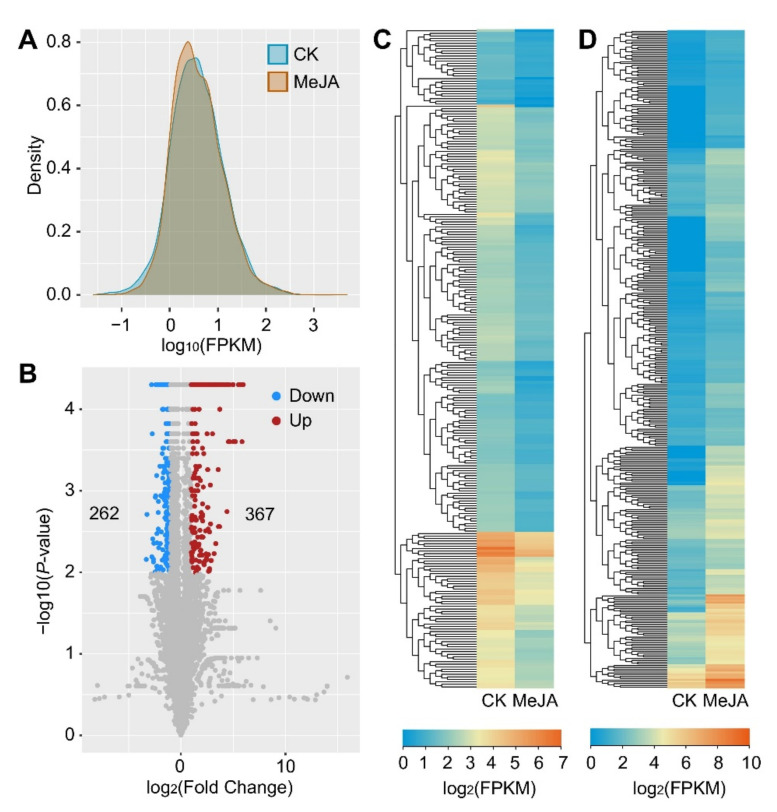
Differential expression analysis of MeJA-responsive lncRNAs in tobacco BY-2 cells. (**A**) Density plot of control and MeJA-treated BY-2 cell samples. (**B**) Volcano plots exploring the relationship between the fold change and the significance of finding MeJA-responsive lncRNAs (*p* value ≤ 0.01; |log_2_FC| ≥ 1). (**C**) Heatmap representing 262 downregulated MeJA-responsive lncRNAs. (**D**) Heatmap representing 367 upregulated MeJA-responsive lncRNAs.

First, we used FPKM values from cufflinks and CummeRbund to evaluate the expression distribution of 7848 lncRNAs in two treatment groups, the CK and MeJA treatment. Figure 3A reveals that the FPKM values of lncRNAs in the CK- and MeJA-treated groups had distinct density distributions, with the discrepancies in FPKM concentrating in the 1 to 10 range. The expression of the lncRNAs in the CK varied from 0 to 13745.88. TCONS_00015430 (FPKM = 3745.88), TCONS_00091915 (FPKM = 2487.72), and TCONS_00091916 (FPKM = 1473.83) were the top three lncRNAs with a high expression (Supplementary Table S6). In addition, the expression of the lncRNAs in the MeJA-treated group varied from 0 to 4957.32. The top three most highly expressed lncRNAs were TCONS 00015430 (FPKM = 4957.32), TCONS 00091915 (FPKM = 2790.99), and TCONS 00091916 (FPKM = 1161.31). The three lncRNAs described above were always the top three most abundant lncRNAs.

According to the results of the differential expression analysis, 629 significantly differentially expressed (DE) lncRNAs were identified as MeJA-responsive lncRNAs, of which 367 were upregulated and 262 were downregulated (*p* value ≤ 0.01; |log_2_FC| ≥ 1) (Figure 3B and Supplementary Table S7). Figure 3C,D depict the expression pattern heatmaps of MeJA-downregulated and -upregulated lncRNAs, respectively. Notably, all of the MeJA-responsive lncRNAs were either unknown or intergenic lncRNAs. The MeJA-upregulated lncRNAs had an upregulation fold change ranging from 5.94489 to 1.00241 (log_2_FC), with TCONS 0010583 showing the highest upregulation fold change. The downregulation fold change of the MeJA-downregulated lncRNAs varied from 3.31928 to 1.00052 (log_2_FC), with TCONS 00005455 showing the highest downregulation fold change. The expression pattern, member number, and fold change of the MeJA-responsive lncRNAs implied that upregulated lncRNAs constituted the major response group following MeJA treatment in BY-2 cells.

### 2.4. Potential Up- and Downregulated Target Genes of MeJA-Upregulated lncRNAs Are Responsible for Different Biological Functions and Metabolic Patterns

**Figure 4:**
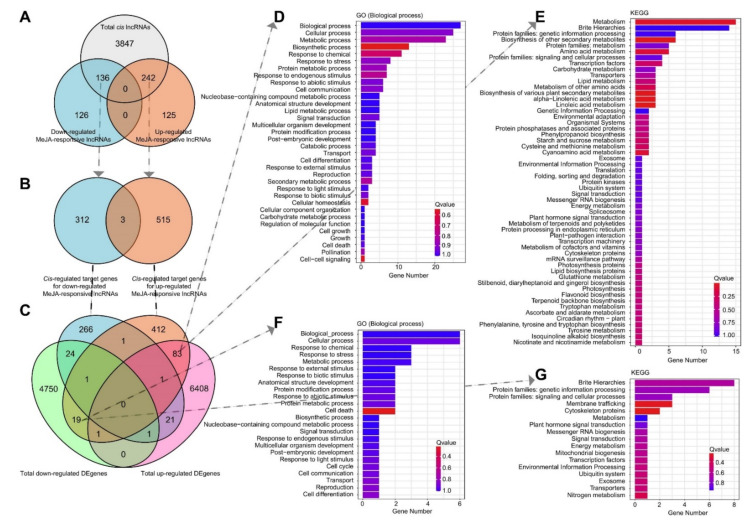
Prediction and functional enrichment of potential *cis*-regulated target genes of MeJA-responsive lncRNAs. (**A**) Numbers of up- and downregulated *cis*-regulated MeJA-responsive lncRNAs. (**B**) Numbers of *cis*-regulated target genes of up- and downregulated MeJA-responsive lncRNAs. (**C**) Expression pattern comparison of *cis*-regulated target genes and MeJA-responsive lncRNAs. (**D**,**E**) Potential biological function enrichment of 83 upregulated *cis*-regulated target genes of upregulated MeJA-responsive lncRNAs. (**F**,**G**) Potential biological function enrichment of 19 downregulated *cis*-regulated target genes of upregulated MeJA-responsive lncRNAs.

Because lncRNAs play vital roles in regulating gene expression, and the potential function of lncRNAs has not been previously reported in tobacco BY-2 cells, identifying and analysing their target genes might aid in our understanding of their activities. We found 9571 lncRNA-mRNA pairs, consisting of 4225 lncRNAs and 7085 adjacent coding genes within 100 kilobases of the lncRNA. Among them, 242 significantly upregulated MeJA-responsive lncRNAs and 136 downregulated MeJA-responsive lncRNAs corresponded to 518 mRNAs and 315 mRNAs via “pairs” of *cis* genes, respectively (Figure 4A,B and Supplementary Table S8). In addition, three mRNAs were associated with one MeJA-up- and downregulated lncRNA (Figure 4B). Among these putative target genes of MeJA-upregulated lncRNAs, 102 genes showed differential expression between the MeJA-treated and control samples, with 83 upregulated and 19 downregulated (Figure 4C).

Interestingly, these two groups of target genes were enriched in significantly different biological functions and metabolic patterns. In the biological process category, 34 GO categories were enriched among the upregulated target genes, whereas 24 GO terms were enriched among the downregulated target genes (Figure 4D,F). In addition, metabolic and biosynthetic process genes were highly prominent, prompting us to focus on metabolic processes. Figure 4E demonstrates that the upregulated target genes for the upregulated MeJA-responsive lncRNAs were significantly enriched in the “metabolism” category (A 09100) (Figure 4E). Moreover, the “environmental adaptation” (B 09159), “metabolism of terpenoids and polyketides” (B 09109), “metabolism of cofactors and vitamins” (B 09108), “metabolism of other amino acids” (B 09106), “amino acid metabolism” (B 09105), and “lipid metabolism” (B 09103) suggested that the upregulated target genes of the MeJA-upregulated lncRNAs initiated plant defence through a string of metabolic processes. However, the downregulated genes were not strongly linked to metabolic activity, and these genes seem to play a role in genetic information processing (Figure 4D,G). This implied that the expression levels of the genes involved in the growth and developmental processes were downregulated by the lncRNAs when the plants were stimulated.

### 2.5. Response-Associated Target Genes of Some Conserved MeJA-Responsive lncRNAs Are Involved in Plant Defence and Stress Resistance

**Figure 5:**
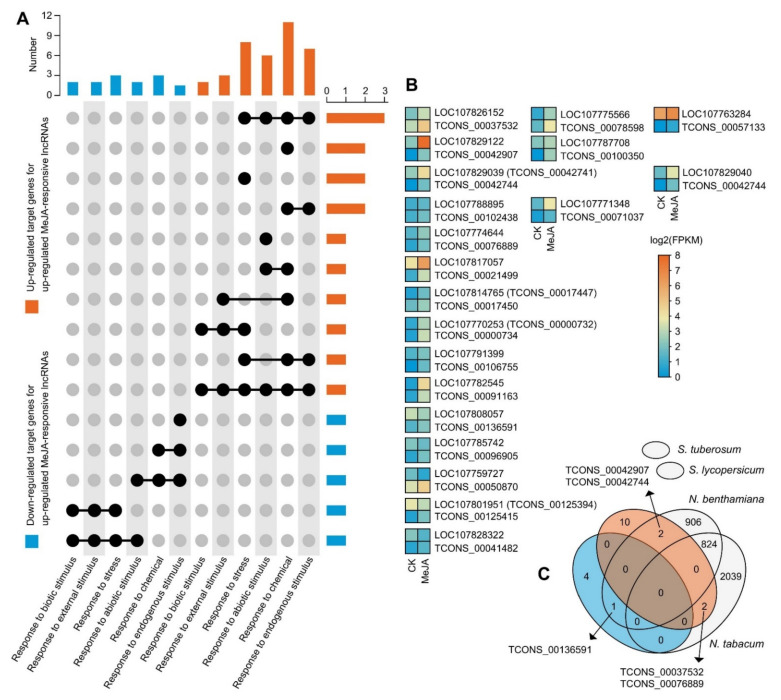
Expression patterns and comparison of *cis*-target genes of MeJA-responsive lncRNAs associated with responsiveness to diverse stimuli. (**A**) Comparison of plant response functions of up- or downregulated target genes of upregulated MeJA-responsive lncRNAs. (**B**) Expression profiles of up- or downregulated target genes and their MeJA-responsive lncRNAs. (**C**) Comparison of homologous upregulated MeJA-responsive lncRNAs with stimulus-response target genes in four Solanaceae plants.

The enrichments in the “response to chemical”, “response to stress”, “response to endogenous stimulus”, “response to abiotic stimulus”, “response to biotic stimulus”, and “response to external stimulus” suggested that the up- and downregulated target genes of the MeJA-upregulated lncRNAs were related to diverse stimuli and plant defence. Furthermore, the response-associated up- or downregulated target genes did not share any members, and several genes worked on different response processes (Figure 5A). We also depicted the expression patterns of all the response-associated target genes and their respective lncRNAs (Figure 5B, Supplementary Table S9). We found a series of target genes that were significantly upregulated and associated with plant resistance to insects and diseases, such as linoleate 9S-lipoxygenase, which plays a role in plant-pathogen interactions (*LOC107770253*), and 21 kDa seed protein-like with endopeptidase inhibitor activity (*LOC107782545*) (Figure 5) [41,42]. In Solanaceae, we found the lncRNAs homologous or paralogous to *N. tabacum* and *N. benthamiana* in the BY-2 cells and speculated that the target genes of these conserved lncRNAs might have their own specificity. Among them, the upregulated MeJA-responsive lncRNAs with upregulated target genes were TCONS_00042907, TCONS_00042744, TCONS_00037532, and TCONS_00076889, and the upregulated lncRNA with downregulated target genes was TCONS_00136591 (Figure 5C). Specifically, TCONS_00042907 targeted *LOC107829122*, which encodes a quinolinate phosphoribosyltransferase (QPT) family member [43]. We discovered that the transcript levels of *LOC107829122* rose approximately 50-fold and that the expression of TCONS 00042907 was detectable exclusively in the MeJA-treated group (Figure 5B, Supplementary Table S9). In addition, *LOC107826152* was the target gene of TCONS_00037532 and encoded the E3 ubiquitin–protein ligase RMA1H1-like, which has been associated with drought stress [44]. However, the high-affinity nitrate transporter 2.4-like (*LOC107808057*) was the only downregulated conserved target gene corresponding to the upregulated lncRNAs. In summary, the response-associated target genes of conserved MeJA-responsive lncRNAs might be involved in plant defence and stress resistance.

### 2.6. MeJA-Responsive Stress- or Defence-Related Cis-Regulatory Elements May Be Responsible for the Upregulation of lncRNAs and Be Involved in Plant Defence through Their Target Genes

**Figure 6:**
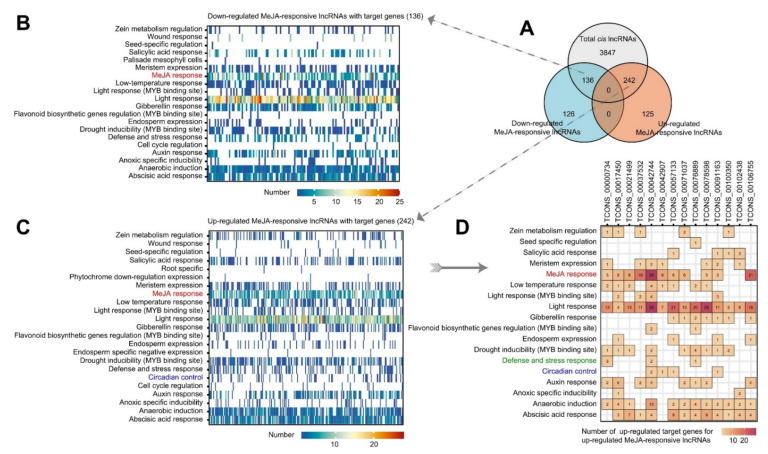
*Cis*-acting element patterns of up- or downregulated MeJA-responsive lncRNAs with *cis*-target genes. (**A**) Numbers of up- and downregulated *cis*-regulated MeJA-responsive lncRNAs. (**B**,**C**) Status of *cis*-acting elements that are present upstream of down- and upregulated MeJA-responsive IncRNAs with target genes. (**D**) Detailed *cis*-acting elements of upregulated MeJA-responsive lncRNAs having upregulated target genes associated with a stimulus-response (Figure 5).

To understand the transcriptional regulation and potential functions of the MeJA-responsive lncRNAs with target genes, the *cis*-elements in the promoter sequence (2000 bp upstream of 5’) were extracted from the tobacco BY-2 cell genome (Supplementary Table S10). As shown in Figure 6, all the MeJA-responsive lncRNAs with target genes possessed many motif elements related to the light response. The 242 downregulated MeJA-responsive lncRNAs with target genes shared 19 similar functional *cis*-acting elements, with 136 downregulated MeJA-responsive lncRNAs having target genes, among which “MeJA response”, “abscisic acid response”, “auxin response”, and “anaerobic induction” stood out (Figure 6B,C). It was not surprising that MeJA-associated *cis*-acting elements, the TGACG-motif and CGTCA-motif, appeared in the upstream sequences of the MeJA-responsive lncRNAs. Only the upregulated MeJA-responsive lncRNAs with the target genes had circadian control elements (the CAAAGATATC-motif), which was likely because the MeJA treatment caused some lncRNAs to be upregulated and interfered with the normal rhythmic activity of the plant. After analysing the response-related upregulated target genes for the upregulated MeJA-responsive lncRNAs with *cis*-acting elements, we found many MeJA-responsive elements upstream of TCONS_00042744 and TCONS_00042907 (Figure 6D). The target gene of TCONS_00037532 is the E3 ubiquitin–protein ligase RMA1H1-like that is associated with plant drought stress, and the target gene of TCONS_00042744 (*LOC107829039*) encodes an isoform of the lipoxygenase-6 protein, which plays an essential role in the plant’s response to pathogen and pest attack. Interestingly, the *cis*-elements of the “circadian control” and “defence and stress response” appeared upstream of TCONS_00042744, which probably conferred the disease resistance to the corresponding target genes. We hypothesized that after the MeJA treatment, the lncRNAs might be activated by their upstream MeJA-responsive *cis*-acting elements. Again, the presence of plant defence-related elements allowed the lncRNAs and their target genes to be involved in plant disease resistance.

## 3. Discussion

A large number of lncRNAs are associated with plant defence, but there have been no reports about them in tobacco BY-2 cells after MeJA treatment. Tobacco BY-2 cells, which were established from a callus, have been considered to be the “HeLa” cell line in higher plant cell biology [45]. MeJA has been used in experiments to mimic herbivory and induce plant defences, and therefore BY-2 cells treated with MeJA helped us to accurately study the lncRNAs involved in plant defence [28]. In this study, we systematically found a total of 7848 potential lncRNAs in tobacco BY-2 cells, using the available tobacco genome from a transcriptomic perspective (Figure 1). The number of identified lncRNAs was smaller than that identified in axillary tobacco buds (13694) and was more significant than that found in tobacco roots (7423), which might be partly due to the highly tissue-specific and cell type-specific lncRNAs [46,47]. Many studies have concluded that lncRNAs vary widely across species, tissues, and cell types, indicating that most are not conserved at the sequence level [48,49]. The low sequence identity among different plants is consistent with previous findings (Figure 2) [50]. In addition, we also found that lncRNAs in *Nicotiana* likely have the same evolutionary origin, suggesting that homologous lncRNAs likely share similar biological functions.

When animals or insects attack plants, the plants produce MeJA and initiate a number of plant defence mechanisms, such as deterring pest-landing, preventing feeding, and reducing palatability [15,16,17]. The MeJA-responsive lncRNAs that were significantly upregulated after the MeJA treatment were the most dominant class, which was likely regulated by MeJA-responsive *cis*-acting elements upstream of the lncRNAs. The TGACG motif and CGTCA motif were the main classes of the MeJA-responsive *cis*-elements (Supplementary Table S10). The tandem TGACG motifs can drive the expression of the 35S promoter in the roots and show responsiveness to signals, such as jasmonic acid, the auxin-related herbicide 2,4-dichlorophenoxyacetic acid, and hydrogen peroxide associated with biotic and abiotic stress [51,52]. In addition, the TGA TFs, the basic leucine zipper subfamily members associated with disease resistance and development, have been discovered to bind to the TGACG motif, and some TGA TFs from *A. thaliana* were shown to be necessary for the activation of jasmonic acid/ethylene-induced defence responses [53,54,55].

After ancestral hybridization, *N. tabacum* eventually became an allotetraploid. The biological reasons for its stable existence must be heterozygous dominance and its derivation of a more powerful gene function and regulatory network [38,39]. Previously, we have explored a series of transcriptional regulators and the JA zinc finger expressed in inflorescence meristem domain proteins associated with alkaloid formation [56,57]. It has been confirmed that lncRNAs are typically found near the genes they regulate, which is known as a *cis*-acting mechanism [48]. It is important to note that the lncRNAs can regulate some *cis*-coding genes, but they could also be coding genes that act in trans. *Trans*-acting lncRNAs, like mRNAs, are transcribed, processed, and then depart their transcription locations to carry out their role somewhere else [58,59]. Here, we focus only on the molecular functions that may result from *cis*-action. In our research, 242 DE upregulated MeJA-responsive lncRNAs, and 136 DE downregulated MeJA-responsive lncRNAs corresponding to 518 PCGs and 315 PCGs, respectively, within a genomic region of 100 kb, were coexpressed (Figure 4, Supplementary Table S8). Furthermore, 20 of them were associated with the GO term “response to chemical, stress, endogenous stimulus, abiotic stimulus, biotic stimulus, and external stimulus” (Figure 5), suggesting that these lncRNAs may play vital roles in plant defence by modulating their target genes. Indeed, the presumably largest group of *cis*-acting lncRNAs are those that function to strengthen target gene expression, similar to how enhancers work [60]. The potential activation ability of most MeJA-responsive lncRNAs on the target genes was also shown by comparing expression patterns (Figure 5). For example, TCONS_00042907 targeted *LOC107829122*, which encodes a QPT family member, and the transcript levels of *LOC107829122* rose approximately 50-fold. The expression of TCONS_00042907 was detectable exclusively in the MeJA-treated group (Figure 5B, Supplementary Table S9). In topping-treated tobacco, the enhancement of nicotine biosynthesis and the upregulation of QPT2 are achieved by the *trans*-acting lncRNA (nta-eTMX27)-mediated inhibition of the miRNA expression [61]. This result provides another lncRNA case for the regulation of QPT from the *cis*-acting perspective.

QPTs are the rate-limiting steps of plant nicotine synthesis, and the importance of nicotine for plant defence cannot be overstated [14,62]. In addition, we also described other target genes of MeJA-responsive lncRNAs that are relevant to plant resistance to stimuli. The action of lipoxygenases (9-LOX and 13-LOX) or α-dioxygenase (α-DOX) initiates the upstream biosynthesis of plant JA, and LOX-derived oxylipins can be involved in the pathogen infection of plants [63]. In cultured potato cells treated with an elicitor that causes late blight disease, there was an accumulation of 9-LOX transcripts, which indicated plant defences [64]. In our analysis, *LOC107829039* and *LOC107770253* are members of the lipoxygenase family. In addition, *LOC107774644* might encode an isoform of the MADS-box TFs involved in stress responses, and a MADS-box TF in *Capsicum annuum* was induced by MeJA [64]. *LOC107829039*, *LOC107770253*, and *LOC107774644* all have *cis*-acting elements upstream of their respective lncRNAs associated with plant defence and stress (Figure 6). Therefore, we hypothesized that MeJA was produced after plant stress and that lncRNAs may be activated by their upstream MeJA-responsive *cis*-acting elements. Subsequently, the presence of plant defence-related elements allows lncRNAs and their target genes to engage in plant disease resistance. Moreover, many genes encoding the transcription factors have also become targets of MeJA-responsive lncRNAs, such as RAX3-like (*LOC107785742*), RAX1-like (*LOC107788895*), myb-related protein 306-like (*LOC107775566*), and MADS-box 23-like isoform X1 (*LOC107774644*). In summary, these TFs are highly likely to regulate MeJA-responsive lncRNAs and their target genes at the transcriptional level.

## 4. Materials and Methods

### 4.1. Plant Materials and Growth Conditions

Suspension cultures of *Nicotiana tabacum* L. cv. Bright Yellow-2 (BY-2) cells [65] were grown and maintained, according to that which was previously reported [57]. Briefly, BY-2 cells were cultured in a 50-mL half-strength Murashige and Skoog (MS) medium (pH 5.8), supplied with 3% (*w*/*v*) sucrose and 0.2 mg/L of 2,4-dichlorophenyoxyacetic acid (2,4-D) in a 250-mL flask, shook with 150 rpm at 28 °C, and subcultured in a fresh medium for every week. For the MeJA treatment, a four-day cell suspension was subcultured in a fresh medium without 2,4-D for one day, then treated with MeJA at a final concentration of 100 μM [dissolved in dimethyl sulfoxide (DMSO) and sterilised with a 0.22-µm Millex syringe filter] for 2 h. Cells treated with an equal volume of DMSO were used as a mock treatment. Vacuum filtration was used to collect cells from the treated cultures for further analysis.

### 4.2. General RNA-Seq Read Mapping and Assembly

The paired-end transcriptome sequencing data of BY-2 cells were obtained from the NCBI Sequence Read Archive (SRA: SRP026451). The general RNA-seq processing pipeline is shown in the workflow (Figure 1A). We used FastQC (http://www.bioinformatics.babraham.ac.uk/projects/download.html#fastqc) (version 0.11.9) (the Babraham Institute, Cambridge, UK) and a FastX-Toolkit (hannonlab.cshl.edu/fastx toolkit/) (version 0.0.14) (Cold Spring Harbor Laboratory, Laurel Hollow, NY, USA) to assess the quality of the initial reads and trimmed the raw data. The minimal quality score to preserve was set to 20, the minimum percent of the bases was set to 90, and the first seven nucleotides were trimmed off. For indexing, the reference genome sequences of *N. tabacum* (GCF 000715135.1) from Bowtie2 (http://sourceforge.net/projects/bowtie-bio/files/bowtie2/2.2.6/) (version 2.2.6) (Johns Hopkins University, Baltimore, MD, USA) were used [66]. TopHat2 (https://ccb.jhu.edu/software/tophat/index.shtml) (version 2.0.14) (University of Maryland, College Park, MD, USA) aligned clean reads to the genome and discovered transcript splice sites (maximum intron size = 5000) [67]. Cufflinks (http://cole-trapnell-lab.github.io/cufflinks/install/) (version 2.2.1) (University of California, Berkeley, CA, USA) were utilized for the transcript assembly, combination, and comparison, in which the transcript expression levels were normalized using fragments per kilobase of transcript per million fragments (FPKM).

### 4.3. lncRNA Identification and Prediction

Several tight screening procedures were used based on the structural properties of lncRNAs and the functional characteristics of nonencoded proteins, as illustrated in Figure 1A. To discover lncRNAs, we preserved transcripts with class codes “i”, “o”, “u”, and “x”, which were most likely noncoding (i, a transfrag falling entirely within a reference intron; o, generic exonic overlap with a reference transcript; u, unknown intergenic transcript; x, exonic overlap with reference on the opposite strand) (http://cole-trapnell-lab.github.io/cufflinks/cuffcompare/) (version 2.2.1) (University of California, Berkeley, CA, USA). In addition, all isoforms with fewer than 200 bp were removed. To analyse transcripts with low coding possibility scores, a coding potential calculator 2 (CPC2) (http://cpc.cbi.pku.edu.cn/) (Peking University, Beijing, China) was employed (scores greater than 0 were eliminated). We used the Pfam database (http://pfam.xfam.org/) (Genome Campus, Hinxton, UK) to help us filter out transcripts with protein-coding domains (E-value < 1 × 10^−5^). Mature miRNAs were blasted against putative lncRNA transcripts, and miRNA precursors were identified and removed (https://www.mirbase.org/ftp.shtml) (The University of Manchester, Manchester, UK). Based on the available tobacco RNA data (https://ftp.ncbi.nlm.nih.gov/genomes/all/GCF/000/715/135/GCF 000715135.1 Ntab-TN90/) (National Library of Medicine, Bethesda, MD, USA), possible mRNAs and ncRNAs were removed using BLAST (E-value < 1 × 10^−5^). Ultimately, using the trustworthy FPKM value (Status = OK) produced by the cufflinks, we obtained reliable putative lncRNAs in *N. tabacum*. The phylogenetic tree of the 39 species was rebuilt using the “Rebuilt Plant PhyloTree” module in TBtools. The conservation of lncRNAs (from green alga to eudicots) was detected via BLASTN (E-value < 1 × 10^−5^). CANTATA (http://rhesus.amu.edu.pl/CANTATA/index.html) (Adam Mickiewicz University, Poznan, Poland) and PlncDB (https://www.tobaccodb.org/plncdb/) (China Tobacco Gene Research Center, Zhengzhou, China) provided available data on 39 species of lncRNAs.

### 4.4. Differential Expression Analysis of MeJA-Responsive lncRNAs

CummeRbund (http://compbio.mit.edu/cummeRbund/) (version 2.7.2) (Massachusetts Institute of Technology, Cambridge, MA, USA), an R software (version 4.1.3) (Lucent Technologies, Murray Hill, NJ, USA), was used to extract gene expression data, differentially expressed genes, gene annotations, and other information from the Cuffdiff output. To calculate the change in expression, the formula fold change (FC) = FPKM of MeJA/FPKM of CK was used. Our study classified genes as DE genes that were differentially expressed after MeJA treatment relative to the control treatment (*p* value ≤ 0.01; |log_2_FC| ≥ 0.5). For lncRNAs, only some lncRNA transcripts with |log_2_FC| values ≥ 1 and a *p* value ≤ 0.01 were deemed MeJA-responsive. All heatmaps with clustering visualized the expression of MeJA-responsive lncRNAs using TBtools software (http://www.tbtools.org/) (version 1.1047) (South China Agricultural University, Guangdong, China) (log scale: base = 2; log with = 1).

### 4.5. Prediction of Cis-Regulating Target Genes of MeJA-Responsive lncRNAs

Based on the *cis*-acting mechanism, we predicted the target genes of all lncRNAs. Potential *cis*-regulating target genes were those transcribed within a 100 kb window upstream or downstream of lncRNAs [68]. Bedtools (https://github.com/arq5x/bedtools2/releases) (version 2.30.0) (The University of Utah, Salt Lake City, UT, USA) was used to find the lncRNAs’ nearby protein-coding genes (PCGs). Venns were utilized to show up- and downregulated MeJA-responsive lncRNAs, *cis*-regulating lncRNAs, and their target genes.

### 4.6. Expression Pattern Comparison of Cis-Regulated Target Genes with Their MeJA-Responsive lncRNAs

We analysed all upregulated DEgenes, all downregulated DEgenes, all *cis*-regulated target genes of upregulated MeJA-responsive lncRNAs, and all *cis*-regulated target genes of downregulated MeJA-responsive lncRNAs using Venn modules from the TBtools software.

### 4.7. Gene Ontology and Pathway Enrichment Analysis

We concentrated on the potential biological functions of *cis*-regulated target genes of MeJA-responsive lncRNAs (including both down- and upregulated target genes). Using an eggNOG-mapper (http://eggnog-mapper.embl.de/) (EMBL), we performed a Gene Ontology (GO) analysis of the protein sequences encoded by these target genes. Using the KEGG (Kyoto Encyclopedia of Genes and Genomes) database (http://www.genome.ad.jp/kegg/) (Kyoto University, Kyoto, Japan), enrichment pathway analysis was performed to investigate the probable activities of the target genes in plant pathways.

### 4.8. Cis-Regulatory Element Prediction in the Promoter Sequences

To understand the *cis*-regulatory element status of the MeJA-responsive lncRNAs, we implemented *cis*-regulatory element analysis with PlantCARE (http://bioinformatics.psb.ugent.be/webtools/plantcare/html/) (Universiteit Gent, Ghent, Belgium). The 2000 bp upstream of the lncRNA gene sequences represented the promoter region.

## 5. Conclusions

In this study, we identified 7848 Nicotiana genus-specific lncRNAs in BY-2 cells, of which 629 differentially expressed (DE) lncRNAs were defined as MeJA-responsive lncRNAs. The combined analysis of the *cis*-regulated lncRNAs and their target genes revealed potential up- and downregulated target functional genes. Additionally, a series of MeJA-responsive lncRNA target genes played a role in nicotine synthesis and disease and insect resistance, which was associated with their MeJA- and defence-related *cis*-regulatory elements. In conclusion, the foregoing outcome indicates that MeJA-responsive lncRNAs regulate nicotine biosynthesis and disease resistance by regulating their potential target genes in BY-2 cells. While these findings reveal a wide range of MeJA-responsive lncRNAs in tobacco BY-2 cells, further research is required to elucidate the molecular mechanisms of coding and noncoding genes at the transcription level and protein level and in epigenetic inheritance. In brief, this work provided novel insights into their plant defence-associated target genes and an abundant gene resource in tobacco for functional research.

## Data Availability

The original contributions presented in the study are included in the article/Supplementary Materials, and further inquiries can be directed to the corresponding author/s.

## Supplementary Materials

The following supporting information can be downloaded at: https://www.mdpi.com/article/10.3390/ijms232415568/s1.

## References

[1] Pauli A., Rinn J.L., Schier A.F. (2011). Non-Coding RNAs as Regulators of Embryogenesis. Nat. Rev. Genet..

[2] Rai M.I., Alam M., Lightfoot D.A., Gurha P., Afzal A.J. (2019). Classification and Experimental Identification of Plant Long Non-Coding RNAs. Genomics.

[3] Yu Y., Zhang Y., Chen X., Chen Y. (2019). Plant Noncoding RNAs: Hidden Players in Development and Stress Responses. Annu. Rev. Cell Dev. Biol..

[4] Sarropoulos I., Marin R., Cardoso-Moreira M., Kaessmann H. (2019). Developmental Dynamics of LncRNAs across Mammalian Organs and Species. Nature.

[5] Chen M., Wang C., Bao H., Chen H., Wang Y. (2016). Genome-Wide Identification and Characterization of Novel LncRNAs in Populus under Nitrogen Deficiency. Mol. Genet. Genom..

[6] Böhmdorfer G., Wierzbicki A.T. (2015). Control of Chromatin Structure by Long Noncoding RNA. Trends Cell Biol..

[7] Wu H., Yang L., Chen L.L. (2017). The Diversity of Long Noncoding RNAs and Their Generation. Trends Genet..

[8] Chen M., Penfield S. (2018). Feedback Regulation of COOLAIR Expression Controls Seed Dormancy and Flowering Time. Science.

[9] Kim D.H., Sung S. (2013). Coordination of the Vernalization Response through a VIN3 and FLC Gene Family Regulatory Network in Arabidopsis. Plant Cell.

[10] Bardou F., Ariel F., Simpson C.G., Romero-Barrios N., Laporte P., Balzergue S., Brown J.W.S., Crespi M. (2014). Long Noncoding RNA Modulates Alternative Splicing Regulators in Arabidopsis. Dev. Cell.

[11] Röhrig H., Schmidt J., Miklashevichs E., Schell J., John M. (2002). Soybean ENOD40 Encodes Two Peptides That Bind to Sucrose Synthase. Proc. Natl. Acad. Sci. USA.

[12] Sousa C., Johansson C., Charon C., Manyani H., Sautter C., Kondorosi A., Crespi M. (2001). Translational and Structural Requirements of the Early Nodulin Gene Enod40, a Short-Open Reading Frame-Containing RNA, for Elicitation of a Cell-Specific Growth Response in the Alfalfa Root Cortex. Mol. Cell Biol..

[13] Begcy K., Dresselhaus T. (2018). Epigenetic Responses to Abiotic Stresses during Reproductive Development in Cereals. Plant Reprod..

[14] Mitchell C., Brennan R.M., Graham J., Karley A.J. (2016). Plant Defense against Herbivorous Pests: Exploiting Resistance and Tolerance Traits for Sustainable Crop Protection. Front. Plant Sci..

[15] Stenberg J.A., Heil M., Åhman I., Björkman C. (2015). Optimizing Crops for Biocontrol of Pests and Disease. Trends Plant Sci..

[16] White C., Eigenbrode S.D. (2000). Effects of Surface Wax Variation in *Pisum sativum* on Herbivorous and Entomophagous Insects in the Field. Environ. Entomol..

[17] Zúñiga G., Corcuera L.J. (2011). Effect of Gramine in the Resistance of Barley Seedlings to the Aphid *Rhopalosiphum* Padi. Entomol. Exp. Appl..

[18] Steppuhn A., Gase K., Krock B., Halitschke R., Baldwin I.T. (2004). Nicotine’s Defensive Function in Nature. PLoS Biol..

[19] Memelink J., Verpoorte R., Kijne J.W. (2001). ORCAnization of Jasmonate-Responsive Gene Expression in Alkaloid Metabolism. Trends Plant Sci..

[20] Kutchan T.M. (1995). Alkaloid Biosynthesis [Mdash] The Basis for Metabolic Engineering of Medicinal Plants. Plant Cell.

[21] Oksman-Caldentey K.M. (2007). Tropane and Nicotine Alkaloid Biosynthesis-Novel Approaches towards Biotechnological Production of Plant-Derived Pharmaceuticals. Curr. Pharm. Biotechnol..

[22] Shoji T., Ogawa T., Hashimoto T. (2008). Jasmonate-Induced Nicotine Formation in Tobacco Is Mediated by Tobacco *COI1* and *JAZ* Genes. Plant Cell Physiol..

[23] Shi Q., Li C., Zhang F. (2006). Nicotine Synthesis in Nicotiana Tabacum L. Induced by Mechanical Wounding Is Regulated by Auxin. J. Exp. Bot..

[24] de Geyter N., Gholami A., Goormachtig S., Goossens A. (2012). Transcriptional Machineries in Jasmonate-Elicited Plant Secondary Metabolism. Trends Plant Sci..

[25] Ryan C.A., Lamb C.J., Jagendorf A.T., Kolattukudy P.E., Doares S.H., Syrovetst T., Weilert E.W. (1995). Oligogalacturonides and Chitosan Activate Plant Defensive Genes through the Octadecanoid Pathway. Proc. Natl. Acad. Sci. USA.

[26] Baldwin I.T., Schmelz E.A., Ohnmeiss T.E. (1994). Wound-Induced Changes in Root and Shoot Jasmonic Acid Pools Correlate with Induced Nicotine Synthesis in Nicotiana Sylvestris Spegazzini and Comes. J. Chem. Ecol..

[27] Creelman R.A., Tierney M.L., Mullet J.E. (1992). Jasmonic Acid/Methyl Jasmonate Accumulate in Wounded Soybean Hypocotyls and Modulate Wound Gene Expression. Proc. Natl. Acad. Sci. USA.

[28] Papazian S., Girdwood T., Wessels B.A., Poelman E.H., Dicke M., Moritz T., Albrectsen B.R. (2019). Leaf Metabolic Signatures Induced by Real and Simulated Herbivory in Black Mustard (*Brassica nigra*). Metabolomics.

[29] Boddu J., Jiang C., Sangar V., Olson T., Peterson T., Chopra S. (2006). Comparative Structural and Functional Characterization of Sorghum and Maize Duplications Containing Orthologous Myb Transcription Regulators of 3-Deoxyflavonoid Biosynthesis. Plant Mol. Biol..

[30] Gao Y., Liu J., Chen Y., Tang H., Wang Y., He Y., Ou Y., Sun X., Wang S., Yao Y. (2018). Tomato SlAN11 Regulates Flavonoid Biosynthesis and Seed Dormancy by Interaction with BHLH Proteins but Not with MYB Proteins. Hort. Res..

[31] vom Endt D., Kijne J.W., Memelink J. (2002). Transcription Factors Controlling Plant Secondary Metabolism: What Regulates the Regulators?. Phytochemistry.

[32] Sears M.T., Zhang H., Rushton P.J., Wu M., Han S., Spano A.J., Timko M.P. (2013). NtERF32: A Non-NIC2 Locus AP2/ERF Transcription Factor Required in Jasmonate-Inducible Nicotine Biosynthesis in Tobacco. Plant Mol. Biol..

[33] Zhu B., Yang Y., Li R., Fu D., Wen L., Luo Y., Zhu H. (2015). RNA Sequencing and Functional Analysis Implicate the Regulatory Role of Long Non-Coding RNAs in Tomato Fruit Ripening. J. Exp. Bot..

[34] de Boer K., Tilleman S., Pauwels L., vanden Bossche R., de Sutter V., Vanderhaeghen R., Hilson P., Hamill J.D., Goossens A. (2011). Apetala2/Ethylene Response Factor and Basic Helix-Loop-Helix Tobacco Transcription Factors Cooperatively Mediate Jasmonate-Elicited Nicotine Biosynthesis. Plant J..

[35] Chen D., Mubeen B., Hasnain A., Rizwan M., Adrees M., Naqvi S.A.H., Iqbal S., Kamran M., El-Sabrout A.M., Elansary H.O. (2022). Role of Promising Secondary Metabolites to Confer Resistance Against Environmental Stresses in Crop Plants: Current Scenario and Future Perspectives. Front. Plant Sci..

[36] Proost S., Pattyn P., Gerats T., van de Peer Y. (2011). Journey through the Past: 150 Million Years of Plant Genome Evolution. Plant J..

[37] Leitch I.J., Hanson L., Lim K.Y., Kovarik A., Chase M.W., Clarkson J.J., Leitch A.R. (2008). The Ups and Downs of Genome Size Evolution in Polyploid Species of *Nicotiana* (Solanaceae). Ann. Bot..

[38] Hochholdinger F., Baldauf J.A. (2018). Current Biology Heterosis in Plants. Curr. Biol..

[39] Mo Z., Luo W., Pi K., Duan L., Wang P., Ke Y., Zeng S., Jia R., Liang T., Huang Y. (2022). Comparative Transcriptome Analysis between Inbred Lines and Hybrids Provides Molecular Insights into K^+^ Content Heterosis of Tobacco (*Nicotiana Tabacum* L.). Front Plant Sci..

[40] Shi C., Zhao L., Zhang X., Lv G., Pan Y., Chen F. (2019). Gene Regulatory Network and Abundant Genetic Variation Play Critical Roles in Heading Stage of Polyploidy Wheat. BMC Plant Biol..

[41] Howe G.A., Schilmiller A.L. (2002). Oxylipin Metabolism in Response to Stress. Curr. Opin. Plant Biol..

[42] Green T.R., Ryan C.A. (1972). Wound-Induced Proteinase Inhibitor in Plant Leaves: A Possible Defense Mechanism against Insects. Science.

[43] Ryan S.M., Cane K.A., DeBoer K.D., Sinclair S.J., Brimblecombe R., Hamill J.D. (2012). Structure and Expression of the Quinolinate Phosphoribosyltransferase (QPT) Gene Family in Nicotiana. Plant Sci..

[44] Lee H.K., Cho S.K., Son O., Xu Z., Hwang I., Kim W.T. (2009). Drought Stress-Induced Rma1H1, a RING Membrane-Anchor E3 Ubiquitin Ligase Homolog, Regulates Aquaporin Levels via Ubiquitination in Transgenic Arabidopsis Plants. Plant Cell.

[45] Nagata T., Nemoto Y., Hasezawa S. (1992). Tobacco BY-2 Cell Line as the “HeLa” Cell in the Cell Biology of Higher Plants. Int. Rev. Cytol..

[46] Chen X., Sun S., Liu F., Shen E., Liu L., Ye C., Xiao B., Timko M.P., Zhu Q.H., Fan L. (2019). A Transcriptomic Profile of Topping Responsive Non-Coding RNAs in Tobacco Roots (*Nicotiana tabacum*). BMC Genom..

[47] Wang L., Gao J., Wang C., Xu Y., Li X., Yang J., Chen K., Kang Y., Wang Y., Cao P. (2022). Comprehensive Analysis of Long Non-Coding RNA Modulates Axillary Bud Development in Tobacco (*Nicotiana tabacum* L.). Front. Plant Sci..

[48] Gil N., Ulitsky I. (2020). Regulation of Gene Expression by *Cis*-Acting Long Non-Coding RNAs. Nat. Rev. Genet..

[49] Johnsson P., Lipovich L., Grandér D., Morris K.V. (2014). Evolutionary Conservation of Long Non-Coding RNAs; Sequence, Structure, Function. Biochim. Biophys. Acta.

[50] Song X., Hu J., Wu T., Yang Q., Feng X., Lin H., Feng S., Cui C., Yu Y., Zhou R. (2021). Comparative Analysis of Long Noncoding RNAs in Angiosperms and Characterization of Long Noncoding RNAs in Response to Heat Stress in Chinese Cabbage. Hort. Res..

[51] Benfey P.N., Chua N.H. (1990). The Cauliflower Mosaic Virus 35S Promoter: Combinatorial Regulation of Transcription in Plants. Science.

[52] Wang Y., Salasini B.C., Khan M., Devi B., Bush M., Subramaniam R., Hepworth S.R. (2019). Clade I TGACG-Motif Binding Basic Leucine Zipper Transcription Factors Mediate Blade-on-Petiole-Dependent Regulation of Development. Plant Physiol..

[53] Idrovo Espín F.M., Peraza-Echeverria S., Fuentes G., Santamaría J.M. (2012). In Silico Cloning and Characterization of the TGA (Tgacg Motif-Binding Factor) Transcription Factors Subfamily in *Carica papaya*. Plant Physiol. Biochem..

[54] Zhang J.-Y., Qu S.-C., Du X.-L., Qiao Y.-S., Cai B.-H., Guo Z.-R., Zhang Z. (2012). Overexpression of the Malus Hupehensis *MhTGA2* Gene, a Novel BZIP Transcription Factor for Increased Tolerance to Salt and Osmotic Stress in Transgenic Tobacco. Int. J. Plant Sci..

[55] Zander M., la Camera S., Lamotte O., Métraux J.P., Gatz C. (2010). *Arabidopsis Thaliana* Class-II TGA Transcription Factors Are Essential Activators of Jasmonic Acid/Ethylene-Induced Defense Responses. Plant J..

[56] Yang Y., Yan P., Yi C., Li W., Chai Y., Fei L., Gao P., Zhao H., Wang Y., Timko M.P. (2017). Transcriptome-Wide Analysis of Jasmonate-Treated BY-2 Cells Reveals New Transcriptional Regulators Associated with Alkaloid Formation in Tobacco. J. Plant Physiol..

[57] Yang Y., Guo J., Yan P., Li Y., Liu K., Gao P., Zhao H., Chen Y., Wang Y., Timko M.P. (2014). Transcriptome Profiling Identified Multiple Jasmonate ZIM-Domain Proteins Involved in the Regulation of Alkaloid Biosynthesis in Tobacco BY-2 Cells. Plant Mol. Biol. Rep..

[58] Andersen R.E., Hong S.J., Lim J.J., Cui M., Harpur B.A., Hwang E., Delgado R.N., Ramos A.D., Liu S.J., Blencowe B.J. (2019). The Long Noncoding RNA Pnky Is a Trans-Acting Regulator of Cortical Development In Vivo. Dev. Cell.

[59] Huarte M., Guttman M., Feldser D., Garber M., Koziol M.J., Kenzelmann-Broz D., Khalil A.M., Zuk O., Amit I., Rabani M. (2010). A Large Intergenic Noncoding RNA Induced by P53 Mediates Global Gene Repression in the P53 Response. Cell.

[60] Ding M., Liu Y., Liao X., Zhan H., Liu Y., Huang W. (2018). Enhancer RNAs (ERNAs): New Insights into Gene Transcription and Disease Treatment. J. Cancer.

[61] Li F., Wang W., Zhao N., Xiao B., Cao P., Wu X., Ye C., Shen E., Qiu J., Zhu Q.H. (2015). Regulation of Nicotine Biosynthesis by an Endogenous Target Mimicry of MicroRNA in Tobacco. Plant Physiol..

[62] Zenkner F.F., Margis-Pinheiro M., Cagliari A. (2019). Nicotine Biosynthesis in Nicotiana: A Metabolic Overview. Tobacco Sci..

[63] Vellosillo T., Martínez M., López M.A., Vicente J., Cascón T., Dolan L., Hamberg M., Castresana C. (2007). Oxylipins Produced by the 9-Lipoxygenase Pathway in *Arabidopsis* Regulate Lateral Root Development and Defense Responses through a Specific Signaling Cascade. Plant Cell.

[64] Göbel C., Feussner I., Schmidt A., Scheel D., Sanchez-Serrano J., Hamberg M., Rosahl S. (2001). Oxylipin Profiling Reveals the Preferential Stimulation of the 9-Lipoxygenase Pathway in Elicitor-Treated Potato Cells. J. Biol. Chem..

[65] Ikeda T., Matsumoto T., Noguchi M. (1976). Effects of Nutritional Factors on the Formation of Ubiquinone by Tobacco Plant Cells in Suspension Culture. Agric. Biol. Chem..

[66] Sierro N., Battey J.N.D., Ouadi S., Bakaher N., Bovet L., Willig A., Goepfert S., Peitsch M.C., Ivanov N.V. (2014). The Tobacco Genome Sequence and Its Comparison with Those of Tomato and Potato. Nat. Commun..

[67] Trapnell C., Roberts A., Goff L., Pertea G., Kim D., Kelley D.R., Pimentel H., Salzberg S.L., Rinn J.L., Pachter L. (2012). Differential Gene and Transcript Expression Analysis of RNA-Seq Experiments with TopHat and Cufflinks. Nat. Protoc..

[68] Jia H., Osak M., Bogu G.K., Stanton L.W., Johnson R., Lipovich L. (2010). Genome-Wide Computational Identification and Manual Annotation of Human Long Noncoding RNA Genes. RNA.

